# Allotetraploidization in *Brachypodium* May Have Led to the Dominance of One Parent’s Metabolome in Germinating Seeds

**DOI:** 10.3390/cells10040828

**Published:** 2021-04-07

**Authors:** Aleksandra Skalska, Elzbieta Wolny, Manfred Beckmann, John H. Doonan, Robert Hasterok, Luis A. J. Mur

**Affiliations:** 1 Plant Cytogenetics and Molecular Biology Group, Institute of Biology, Biotechnology and Environmental Protection, Faculty of Natural Sciences, University of Silesia in Katowice, 40-032 Katowice, Poland; askalska@us.edu.pl (A.S.); elzbieta.wolny@us.edu.pl (E.W.); 2 Institute of Biological, Environmental and Rural Sciences, Aberystwyth University, Edward Llwyd Building, Aberystwyth SY23 3DA, UK; meb@aber.ac.uk; 3 National Plant Phenomics Centre, Institute of Biological, Environmental and Rural Sciences (IBERS), Aberystwyth University, Aberystwyth SY23 3EE, UK; jhd2@aber.ac.uk; 4 College of Agronomy, Shanxi Agricultural University, Taiyuan 030801, China

**Keywords:** *Brachypodium* species, metabolites, model grass, seed germination

## Abstract

Seed germination is a complex process during which a mature seed resumes metabolic activity to prepare for seedling growth. In this study, we performed a comparative metabolomic analysis of the embryo and endosperm using the community standard lines of three annual *Brachypodium* species, i.e., *B. distachyon* (Bd) and *B. stacei* (Bs) and their natural allotetraploid *B. hybridum* (BdBs) that has wider ecological range than the other two species. We explored how far the metabolomic impact of allotetraploidization would be observable as over-lapping changes at 4, 12, and 24 h after imbibition (HAI) with water when germination was initiated. Metabolic changes during germination were more prominent in *Brachypodium* embryos than in the endosperm. The embryo and endosperm metabolomes of Bs and BdBs were similar, and those of Bd were distinctive. The Bs and BdBs embryos showed increased levels of sugars and the tricarboxylic acid cycle compared to Bd, which could have been indicative of better nutrient mobilization from the endosperm. Bs and BdBs also showed higher oxalate levels that could aid nutrient transfer through altered cellular events. In *Brachypodium* endosperm, the thick cell wall, in addition to starch, has been suggested to be a source of nutrients to the embryo. Metabolites indicative of sugar metabolism in the endosperm of all three species were not prominent, suggesting that mobilization mostly occurred prior to 4 HAI. Hydroxycinnamic and monolignol changes in Bs and BdBs were consistent with cell wall remodeling that arose following the release of nutrients to the respective embryos. Amino acid changes in both the embryo and endosperm were broadly consistent across the species. Taking our data together, the formation of BdBs may have maintained much of the Bs metabolome in both the embryo and endosperm during the early stages of germination. In the embryo, this conserved Bs metabolome appeared to include an elevated sugar metabolism that played a vital role in germination. If these observations are confirmed in the future with more *Brachypodium* accessions, it would substantiate the dominance of the Bs metabolome in BdBs allotetraploidization and the use of metabolomics to suggest important adaptive changes.

## 1. Introduction

Polyploidy is an often observed phenomenon in eukaryotes, resulting in important ecological and evolutionary processes. Many plant lineages show evidence of palaeopolyploidization in their genomes. Polyploid plants usually have larger cell sizes and faster growth, which is very significant from an evolutionary standpoint [1,2]. Polyploidization leads to a series of genomic, metabolomic, cellular, and physiological changes. *Arabidopsis thaliana* autotetraploids have shown different metabolic profiles, indicating that polyploidization affects metabolite accumulation [3]. Similarly, polyploidization in *Hylocereus* and *Citrus* species induced changes in metabolite accumulation patterns [4,5,6]. Thus, polyploidization is a major force in the evolution of both wild and cultivated plants for its ability to confer high environmental adaptability [7].

*Brachypodium* is one of the most studied genera of monocotyledonous plants, mainly due to *Brachypodium distachyon* being a model organism for temperate cereals and other economically important grasses [8,9,10,11,12,13,14]. *B. distachyon* has been used to investigate various aspects of grass biology [15], including cell wall composition [16,17,18,19], host–pathogen interactions [20,21], responses to abiotic stress [22,23,24], the regulation of seed dormancy [25], and seed to seedling transition [26,27]. The genus consists of ~20 species that are distributed worldwide. Two of the diploid annual species, *B. distachyon* (Bd) and *Brachypodium stacei* (Bs) have hybridized to produce an allotetraploid, *Brachypodium hybridum* (BdBs) [28]. BdBs has been proposed as a model system to study polyploidization and grass speciation [29]. It is more widely distributed compared to its progenitors, especially Bs, and tends to have a greater number of seeds per inflorescence and 1000 grain weights. Therefore, allotetraploidization seems to have led to the formation of a species with increased fitness across a range of ecological niches [30,31].

Seed germination is a fundamental step for development, and is accompanied by substantial physiological and biochemical changes that lead to morphological events. Germination is controlled by multiple environmental cues such as light and moisture, and various endogenous factors, including phytohormones. Germination begins with the uptake of water and can be sub-divided into three phases: phase I, where the seed rapidly imbibes water, phase II sees the activation of metabolism, and in phase III, changes occur that lead to hypocotyl emergence [8]. Crucial physiological and biochemical processes such as the hydrolysis of storage compounds, protein biosynthesis, respiration, and cell elongation are activated in the phase II. These metabolic changes are associated with the catabolism of seed storage products into simpler chemical forms, such as sugars and amino acids, that are needed for protein synthesis and bioenergy in the growing seedling [8,9]. Distinct and time-dependent alternations in metabolism have been described for germinating seeds in cereals such as rice [10] and wheat [11], as well as in *A. thaliana* [12]. In the first hour of imbibition, rapid changes in metabolism, including increases in hexose phosphates and tricarboxylic acid cycle intermediates, occur. This phase is also typified by significant reductions in the majority of different metabolites that had previously accumulated during seed desiccation. For example, proteins are degraded by proteases into amino acids during germination [13]. These primary metabolites are rapidly consumed to support a metabolic switch towards biosynthetic processes that are needed for early germination. Later changes in the metabolome during germination include an increase in levels of compounds such as amino acids, sugars, and organic acids to feed hypocotyl growth [12,14].

*Brachypodium* species represent a good model system to assess the possible effects of polyploidization on the germinating seed metabolome. Previous metabolic studies on *Brachypodium* species have focused on different growth stages and different stress conditions, particularly drought [22,32,33] and the genotypic variation for seed metabolite levels [34]. Our initial characterization of a Bd allowed comparison with what is known in wheat [35]. This comparison suggested important differences in the extent of endosperm development. Although similar, key grain development marker genes (histone H4, C13 endopeptidase, pyruvate orthophosphate dikinase (PPDK), α-galactosidase, and globulins 1 and 2) are expressed in both wheat and Bd, their timing appeared to be different [35]. Striking morphological differences include smaller numbers and sizes of starch grains in Bd compared to wheat while endosperm cell walls were approximately twice as thick in Bd as in wheat [35,36]. Additionally, the Bd cell walls become less well-stained by calcofluor white during germination, suggesting a loss of 1-3 β and 1-4 β linked polysaccharides. Thus, the cell wall could be a source of sugars during germination. However, infrared fluorescence imaging suggested that the Bd cell wall was relatively deficient in β-glucans but potentially enriched in hemicelluloses [36].

To provide information on the metabolite profiles during seed germination in *Brachypodium*, we employed flow-infusion high-resolution electrospray mass spectroscopy (FIE–HRMS) to describe the metabolomes of the embryo and endosperm over a 24-h period over imbibition. We tested the hypothesis that the allotetraploidization process may have led to overlapping metabolomes in the three Brachypodium annuals (Bd, Bs, and BdBs). However, our data indicated that the Bs but not the Bd metabolome had considerable similarities with that of BdBs during germination. Further, these Bs/BdBs overlapping metabolomes indicated elevated sugar metabolism that could represent a source of increased fitness during the germination process.

## 2. Materials and Methods

### 2.1. Plant Material and Seed Germination

**Figure 1:**
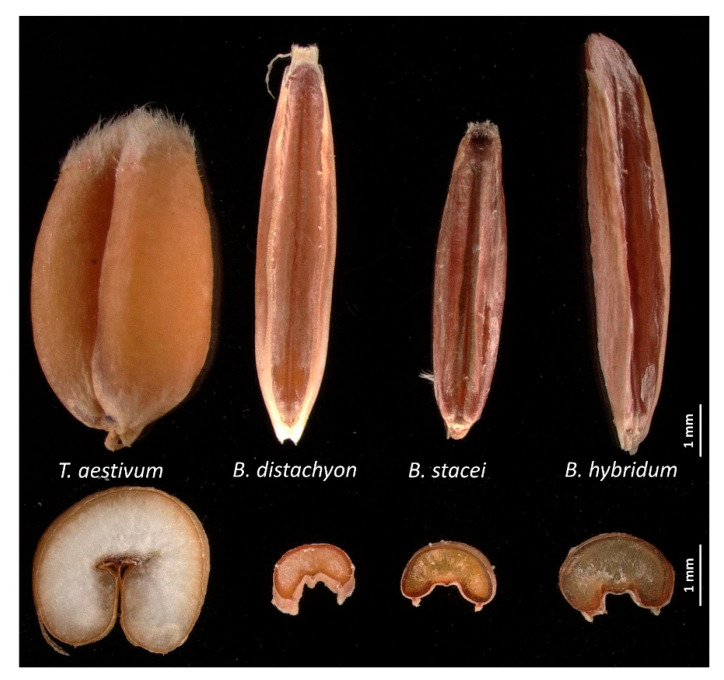
Grains of *Brachypodium* species *Brachypodium distachyon* Bd21 (Bd), *Brachypodium stacei* ABR114 (Bs) and *Brachypodium hybridum* ABR113 (BdBs) compared to wheat (*Triticum aestivum*). Images were from (top) grains where the lemma had been removed and (bottom) transversely sections from seeds. Note the large starchy endosperm in wheat that is greatly diminished in each *Brachypodium* species. Scale bars represent 1 mm.

Seeds of *B. distachyon* Bd21 (Bd), *B. stacei* ABR114 (Bs) and *B. hybridum* ABR113 (BdBs) were obtained from the US Department of Agriculture, National Plant Germplasm System and the Institute of Biological, Environmental and Rural Sciences, Aberystwyth University, UK. The lemma was removed from the seeds to exclude its impact on the germination rate. The general features of the seeds from the different species are shown in Figure 1.

Approximately 500 grains of each *Brachypodium* species were placed on three layers of filter paper that had been soaked with distilled water in Petri dishes and germinated at 22–24 °C in the dark. The grains were collected at 4, 12, and 24 h after imbibition (HAI) with five biological replicates. The embryos were isolated, separated from the endosperm, and frozen in the liquid nitrogen.

### 2.2. Sample Preparation and Metabolite Extraction

For metabolomic analyses, frozen embryos and endosperm were extracted using a single-phase extraction solution (chloroform/methanol/water, 1/2.5/1, *v*/*v*/*v*). The collected samples (40 mg each) of frozen embryos and endosperm were homogenized and mixed with 1 mL of extraction solution for 20 min at 4 °C. Samples were centrifuged for 30 min at 4 °C, and the supernatant was transferred to new tubes, from which 200 μL were taken for further analyses. Metabolite fingerprinting was performed using flow-infusion high-resolution electrospray mass spectroscopy (FIE–HRMS) using a Q Exactive Plus mass analyzer instrument with a Dionex U300 Ultra High Performance Liquid Chromatography (UHPLC) system (Thermo Fisher Scientific, Bremen, Germany). Mass-ions (*m/z*) were generated in positive and negative ionization modes in a single run, as described by Baptista et al. [37]. The data are provided in Supplementary Materials Table S1.

### 2.3. Metabolomic Data Analysis

Multivariate analyses were performed with MetaboAnalyst (https://www.metaboanalyst.ca/, accessed on 28 February 2021). Differences in the metabolomic profiles of samples were analyzed with unsupervised principal component analysis (PCA) and supervised partial least squares-discriminant analysis (PLS-DA). The significance of the cross-validated *p*-values were based on one-way ANOVA with Bonferroni correction for false discovery rates (FDR). Multiple comparison and post hoc analyses used Tukey’s honestly significant difference (Tukey’s HSD). The relative contributions of experimental variations (“effects estimates”—in this case, genotype and time) to the total variation used ANOVA–simultaneous component analysis (ASCA), a form of multivariate ANOVA. For each m/z, annotation was made using a 5 ppm tolerance on their accurate mass. Metabolomic annotation was made using DIMEdb (https://dimedb.ibers.aber.ac.uk, accessed on 28 February 2021). Identification was based on the MS peaks to pathway algorithm [37] (tolerance = 5 ppm, reference library; *Oryza sativa*). This involved metabolites being annotated using the Kyoto Encyclopedia of Genes and Genomes (KEGG) database, considering the following possible adducts: [M+]^+^, [M+H]^+^, [M+NH4]^+^, [M+Na]^+^, [M+K]^+^, [M-NH2+H]^+^, [M-CO2H+H]^+^, [M-H2O+H]^+^; [M−]^−^, [M−H]^−^, [M+Na−2H]^−^, [M+Cl]^−^, and [M+K−2H]^−^. Correlations between multiple adducts of a metabolite were used in the identification process.

Metabolite Set Enrichment Analysis (MSEA) and pathway analysis were performed to identify biologically meaningful patterns in the metabolome data using the R-based MetaboAnalyst platform [38]. For pathway analysis algorithms, the Fisher’s exact test was used with a KEGG pathway library for Oryza sativa as a model reference. These analyses considered the number of detected metabolites in individual pathways and any alternations between samples and model reference. Pathway significance was based on whether metabolites from a given sample were overrepresented in a given metabolite set, while pathway impact reflected the role of a given metabolite in particular pathway.

## 3. Results

### 3.1. Metabolic Profiling of the Embryo and Endosperm

**Figure 2:**
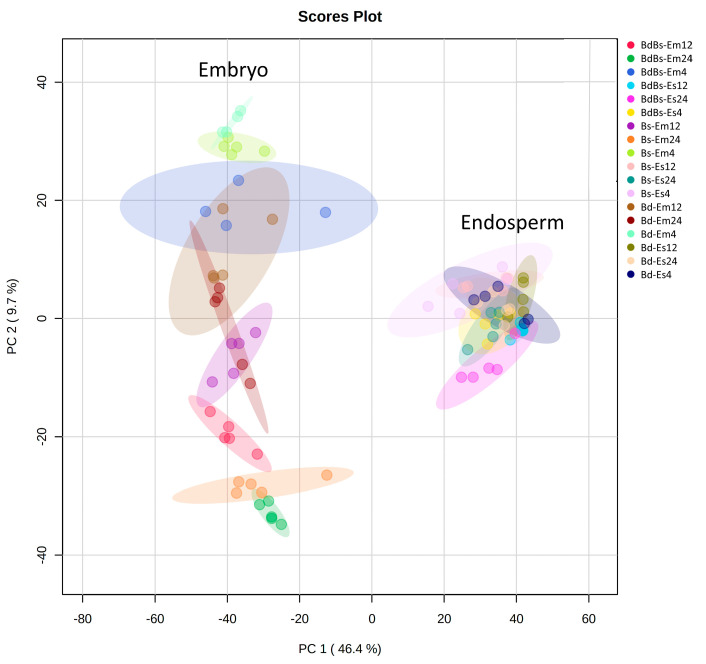
PCA (principal component analysis) of embryo (Em) and endosperm (Es) metabolites in *B. distachyon* Bd21 (Bd), *B. stacei* ABR114 (Bs), and *B. hybridum* ABR113 (BdBs) at 4, 12, and 24 h after imbibition (HAI). PC 1: the first principal component; PC 2: the second principal component.

We performed the metabolic profiling of the embryo and endosperm in Bd, Bs, and BdBs after 4, 12, and 24 HAI. PCA indicated that the major sources of variation were linked to organ type, with separation between embryo and endosperm seen across principal component (PC) 1 (Figure 2). The variation between the embryo samples was higher than that between endosperm samples, as indicated by the extent of variation across PC 2, but species-specific differences were not readily identifiable.

### 3.2. Assessments of Embryo and Endosperm Metabolomes

**Figure 3:**
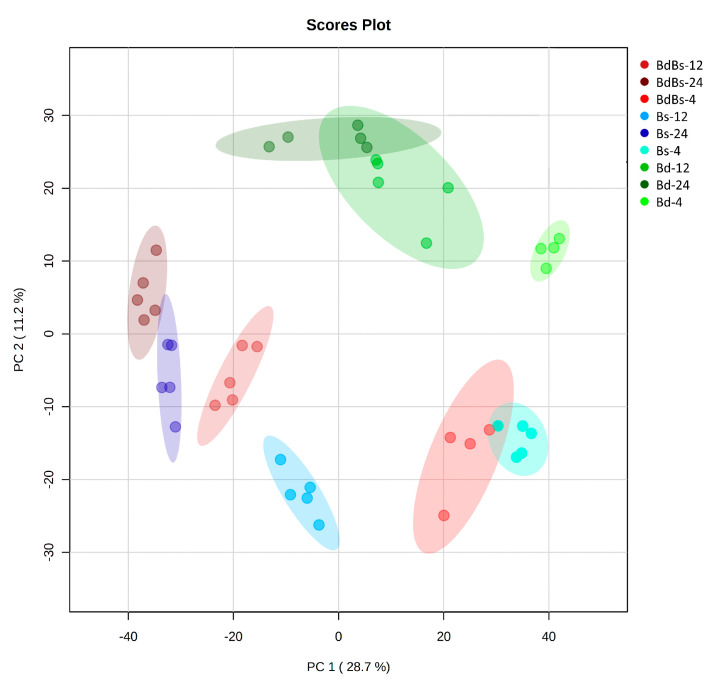
PCA of embryo metabolites in *B. distachyon* Bd21 (Bd), *B. stacei* ABR114 (Bs), and *B. hybridum* ABR113 (BdBs) at 4, 12, and 24 HAI.

To investigate variation within the two different tissue types during imbibition, each was separately assessed. In the embryo samples, the main differences in metabolic profiles were seen with over time (PC 1 = 28.7%), but genotypic differences were also seen (Figure 3). In the case of Bd, the overlap between the 12 and 24 HAI groups suggested that metabolomic changes at these time points were not as pronounced as in Bs and BdBs. Across the axis showing the lesser source of variation (PC 2 = 11.2%), Bd formed a distinct group from the other species.

**Figure 4:**
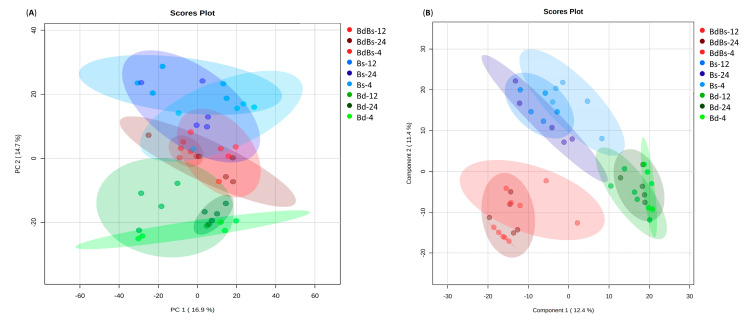
(**A**) PCA and (**B**) partial least squares-discriminant analysis (PLS-DA) of endosperm metabolites in *B. distachyon* Bd21 (Bd), *B. stacei* ABR114 (Bs), and *B. hybridum* ABR113 (BdBs) at 4, 12 and 24 HAI.

Considering the endosperm, no species-specific variation was seen across PC 1, which was instead linked to within experimental class variation. However, some variation that could be linked to species was apparent across PC 2 (PC 2 = 14.7%) (Figure 4A). This suggested that Bd and Bs had distinctive metabolomes but BdBs had some overlap with the Bs group. The use of the supervised PLS-DA allowed for the greater species-specific variation. However, considering only component 1, there was again overlap with Bs and BdBs, with Bd again being the most separate group (Figure 4B). Although the endosperm was being mobilized during this phase, strong metabolomic changes linked to time were not clearly seen. This may reflect the inability of our metabolomic approach to assess changes in starch polymers. Taking all our observations together indicated that the metabolomes of all three species were distinctive but Bs makes the greater metabolic contribution to the allotetraploid BdBs.

### 3.3. Species Specific Metabolite Variation

**Figure 5:**
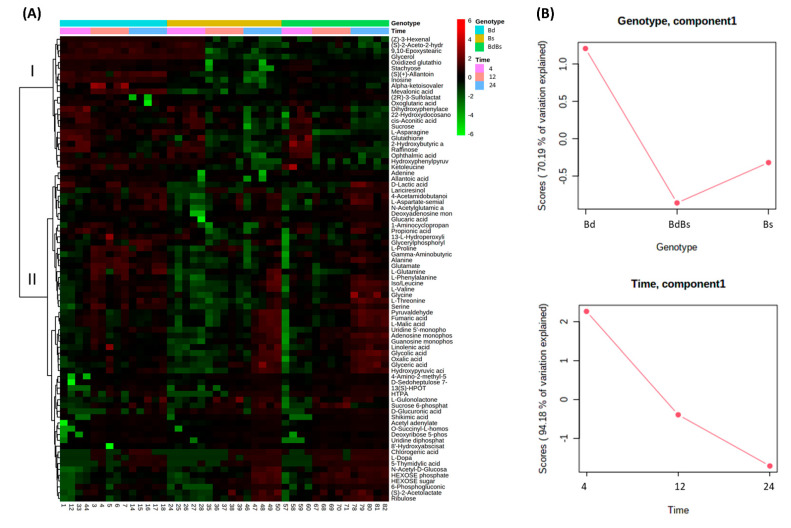
Metabolomic variation in *Brachypodium* embryos during germination. (**A**) Heatmap of major sources of metabolite variation and (**B**) ANOVA–simultaneous component analysis showing effect estimates (genotype and time) in *B. distachyon* Bd21 (Bd), *B. stacei* ABR114 (Bs), and *B. hybridum* ABR113 (BdBs) at 4, 12, and 24 HAI.

Next, we considered species-specific changes in each of the *Brachypodium* species in each tissue type occurring over time. These changes were identified based on ANOVA. These species-specific changes were compiled into a single data matrix and compared using a heat map (Figure 5). The dendrogram with the heatmap indicated two major clades, which are labelled “I” and “II” in Figure 5A. In II, the metabolite changes were broadly similar in each species over the 24 HAI, but with I, the changes appeared to be more distinctive in Bd compared to Bs and BdBs. The use of ASCA indicated that within the “genotype” effect, Bd-linked metabolites were the major source of variation, with Bs and BdBs being more similar (Figure 5B). For “time” effects, 4 HAI was the greater source of metabolite variation.

**Figure 6:**
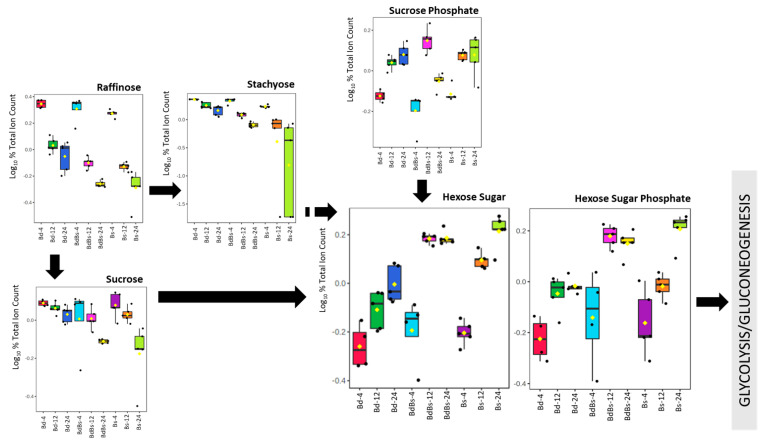
Discriminatory sugar metabolites detected in *Brachypodium* embryos during germination. Metabolites are arranged in accordance with the Kyoto Encyclopedia of Genes and Genomes (KEGG) starch metabolism map (https://www.genome.jp/kegg-bin/show_pathway?map00052+C00492, accessed on 28 February 2021). Data show box and whisker comparison of *B. distachyon* Bd21 (Bd), *B. stacei* ABR114 (Bs), and *B. hybridum* ABR113 (BdBs) at 4, 12, and 24 HAI.

To provide potential functional differences between the *Brachypodium* embryo metabolomes, pathway enrichment analyses were carried based on the key sources of variation within each species (Figure S1). Bd showed a predominance of changes in galactose metabolism that was not so prominent in the other two species. In both Bs and BdBs, the top pathways were dominated by amino acid and glyoxylate metabolism. Some pathways were seen in each species but at differing ranks. These included those linked to bioenergy, starch/sucrose, and nucleotide metabolism, as would be expected during the germination process. To provide some detail of these metabolomic differences, key variables were compared across the species. Considering sugar metabolism leading to glycolysis (Figure 6), raffinose catabolism and increase in imported sucrose-6-phosphate were similar in all species. However, sucrose catabolism appeared to be greater in Bs and BdBs, leading to increases in the detected hexose sugar and hexose sugar phosphates that feed into bioenergetic metabolism. This was reflected in higher levels of pyruvate and TCA metabolites, malate, fumarate and oxaloacetate in Bs and BdBs (Figure S2). Lactate levels tended to be higher in Bd, suggesting relatively less efficient ATP/NADH generation during germination. Bd also saw the greater fall in glutathione and the ratios of chemically reduced glutathione (GSH) vs oxidized glutathione (GSSG) were consistent with the more chemically reducing conditions in Bs and BdBs (Figure S3).

**Figure 7:**
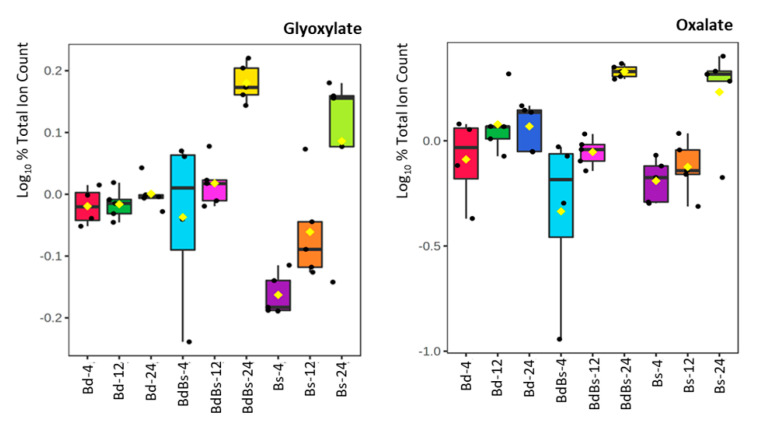
Oxalate metabolites in *Brachypodium* embryos during germination. Data show box and whisker comparison of *B. distachyon* Bd21 (Bd), *B. stacei* ABR114 (Bs), and *B. hybridum* ABR113 (BdBs) at 4, 12, and 24 HAI.

As amino acid metabolism appeared to differ between the species (Figure S1), this was also examined (Figure S4). As expected, the levels of most amino acids appeared to increase with HAI. However, although the changing levels of some amino acids were broadly similar amongst the species (valine, iso/leucine, glycine; Figure S4A), the contents tended to be greater in Bd for most (Figure S4B). Glyoxylate/oxalate metabolism was also ranked highly on the Bs and BdBs enhanced pathway list, and the box and whisker plots of some key metabolites showed that these were increased in those species compared to Bd (Figure 7).

**Figure 8:**
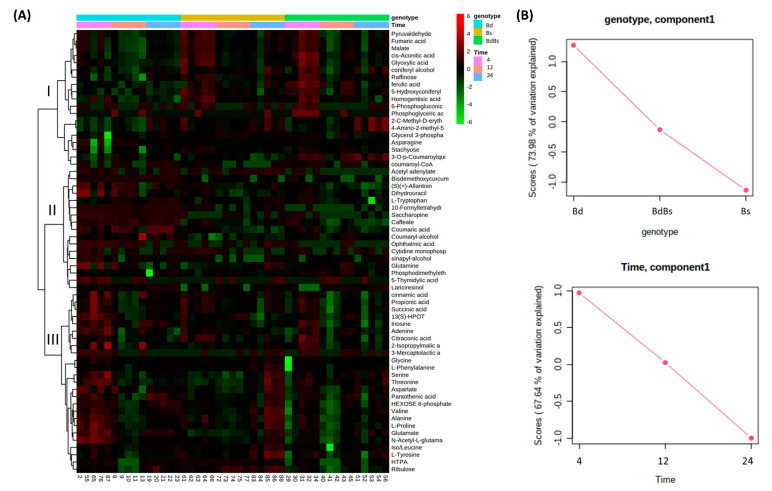
Metabolomic variation in *Brachypodium* endosperm during germination. (**A**) Heatmap of major sources of metabolite variation and (**B**) ANOVA–simultaneous component analysis showing effect estimates (genotype and time) in *B. distachyon* Bd21 (Bd), *B. stacei* ABR114 (Bs), and *B. hybridum* ABR113 (BdBs) at 4, 12, and 24 HAI.

Metabolite changes over time in endosperm for each of the *Brachypodium* species were also identified. These changes were compiled and compared in a heat map (Figure 8A). The dendrogram with the heatmap indicated three major clades, labelled “I,” “II,” and “III” in Figure 8. Clade I contained metabolites that were generally at low levels in Bd during the imbibition period but in Bs and BdBs, after initially high levels at 4 HAI, were reduced with time. In clade II, metabolites were higher in Bd, but they tended not to increase at any time in the other species. In clade III, metabolites were at very high levels in Bd at 4 HAI but were at variable levels in Bs and BdBs. This was consistent with Bd being a major source of variation, as also suggested by ASCA (Figure 8B).

**Figure 9:**
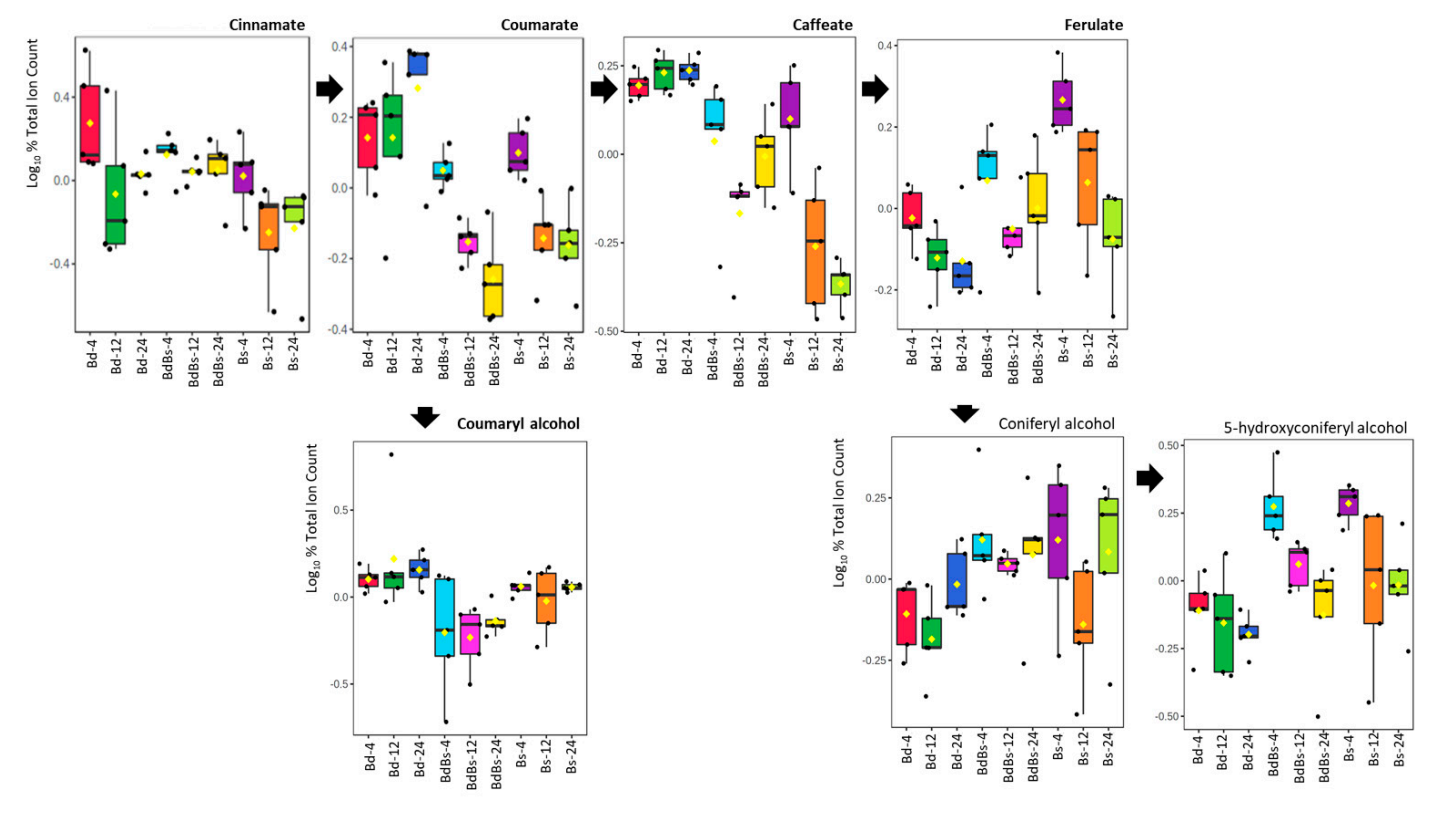
Monolignol metabolites, which are significant sources of variation, in *Brachypodium* endosperm during germination. Data show box and whisker comparison of *B. distachyon* Bd21 (Bd), *B. stacei* ABR114 (Bs), and *B. hybridum* ABR113 (BdBs) at 4, 12, and 24 HAI. The arrangement of the plots reflect the associated between the metabolites, as indicated in https://www.genome.jp/kegg-bin/show_module?M00039 (accessed on 28 February 2021).

To provide functional information, pathway enrichment assessments were undertaken for each species (Figure S5). In each species, metabolites that contributed to the variation were associated with a range of pathways. To ease interpretation, some sources of variation are presented as box and whisker plots. In examining bioenergetic changes compared to embryo, there were fewer sources of variation in the endosperm (Figure S6). Raffinose levels proved to vary over the imbibition period, with each species showing that levels at 4 HAI tended to fall over time. Similarly, pyruvate and the TCA metabolites reduced from high levels at 4 HAI over time. This was consistent with a rapid mobilization of bioenergy-associated metabolites to the embryo. Amino acid metabolism was prominent in the pathway enrichment assessment from each metabolism. Plotting changes in individual amino acids overtime showed various patterns of change (Figure S7). Generally, patterns revealed a reduction in amino acid levels from at 12 HAI from those at 4 HAI, with a recovery in levels at 24 HAI. In Figure 8, we note changes in hydroxycinnamates (caffeate and ferulate) and monolignol metabolites. These were plotted because they could reflect cell wall changes through processing as a source of nutrients [35] (Figure 9). Over time, Bd showed increases in coumarate, caffeate, and (marginally) coumaryl and coniferyl alcohols, which were likely to reflect changes occurring at the cell wall. However, in the cases of ferulate, coniferyl, and 5-hydroxyconferyl alcohol, the changes in cell wall-associated metabolites were greater in Bs and BdBs.

## 4. Discussion

Seed imbibition and germination represents a key initial stage in plant development, the relative success of which will govern whether a plant will thrive in a given environment. A key aspect of the germination process is the mobilization of stored nutrients from the endosperm to the embryo and include amino acids, lipids, and sugars. As a result, this process particularly lends itself to metabolomic analyses. Our studies of germinating *B. distachyon* seeds have focused on the changes in metabolites that occur phases of active cell cycle [26], global histone modifications, and DNA methylation changes during embryonic and post-embryonic growth [27,39]. Future studies may link the metabolomic changes we describe in this paper with epigenetic and transcriptional changes during germination.

The germination metabolomes have been described in barley and wheat [40] or, in a more focused study, in wheat seeds exhibiting different levels of dormancy [41]. In tomato, metabolomics was used to define biochemical quantitative trait loci (QTL) in *Solanum lycopersicum* and *Solanum pimpinellifolium* recombinant inbreed lines (RIL population linked to germination [42]. A more practical application involved metabolomic approaches to detect the early stages of sprouting in cereal seeds, which is relevant to the grain industry [43].

*Brachypodium distachyon* has been exploited in temperate grass-seed research. For example, like the Triticeae, it displays a gene duplication in high-molecular-weight (HMW) glutenin genes that are important in conferring bread-making properties [44]. Considering its distinct features, some key studies have compared *B. distachyon* seeds versus other grasses and cereals [15,35,36,45]. Briefly, *B. distachyon* seeds are relatively large compared to the size of the entire plant, and the endosperm represents around 75% of the dehulled grain weight, which is nearly equivalent to domesticated grasses (~80%). The aleurone layer appears to lack major transfer tissues, which are used in endosperm filling in major temperate cereals but appears to be similar to rice. The grain has a very high level of proteins—amongst the highest detected in grasses. Most striking are the thick cell walls in the endosperm, and, correspondingly, only 10% of the seed is starch as opposed to 35–40% in non-domesticated wheat species [35]. Instead of starch, it appears that the storage polymers in *B. distachyon* are primarily (1,3; 1,4)-β-glucans, with glucose being the main saccharide monomer with lesser contributions from arabinose and xylose. These have been suggested to be mobilized to provide the nutrients during germination in *B. distachyon* [36].

In this study, we explored the metabolic differences of three *Brachypodium* species at three stages of seed germination by using a comparative metabolomics approach. We focused on the use of community standard lines for each species, namely Bd21 (Bd), ABR114 (Bs), and ABR113 (BdBs). These three species comprise a valuable model to investigate the impact of polyploidization on the organization and evolution of plant genomes, as well as grass biology. Polyploidy has a significant influence on the morphology and physiology of newly formed offspring. Compared with the corresponding diploids, autopolyploids tend to have larger cells, which may result in the enlargement of single organs, such as leaves, flowers, and seeds. Physiological traits such as plant height, growth rate, flowering time, and fertility also can be altered by polyploidization [46,47]. Polyploidization might significantly increase stress tolerance [48,49]. Studies on metabolic changes caused by polyploidization have indicated its role in the induction of considerable changes in primary and secondary metabolite accumulation in induced tetraploids compared to their diploid parents [4,5,6]. Similarly, metabolite changes were found in intergeneric hybrids between *Brassica rapa* and *Raphanus sativus* [50]. However, the effect of polyploidization on metabolic changes during seed germination needs to be more fully characterized. A comparative investigation of seed germination, metabolism, and seedling growth between tetraploid, *Triticum durum* (AABB), and the hexaploid, *T. aestivum* (AABBDD), suggested that each species was distinctive [51].

We separated metabolomic assessments of the endosperm from that of the embryo to discriminate source and sink tissue during germination. Metabolomic changes after imbibition appeared to be greater in the embryo than in the endosperm (Figure 3 vs. Figure 4). This should not be taken to indicate that the endosperm is less metabolically active, as these data could reflect the metabolomic approach that we employed. FIE-HRMS is biased towards small metabolites (<1500 *m/z*) so that changes in large polymers may not be readily captured. It may also be that the breakdown products are rapidly transported out of the endosperm and so were below our detection limits. 

Results from both embryo and endosperm suggest that the Bs and BdBs metabolomes were similar to each other, but that Bd was distinctive. Therefore, in defining the actual metabolite changes occurring on imbibition, it was relevant to consider what features were common to the *Brachypodium* and what elements of the Bs metabolome were conserved in the allotetraploid BdBs.

Sugar metabolism was detected in the embryos of all three species. Each showed that from 4 HAI, the trisaccharide raffinose and sucrose levels were reduced with increasing imbibition time. This was complemented by increases in monosaccharide hexose sugars (“hexose-sugars”; Figure 6) and sugar phosphates, all of which feed into glycolysis/gluconeogenesis. As photosynthesis does not occur during seed germination, the energy for physiological processes and seedling development depends on glycolysis [52], and the availability of phosphorylated sugars play a key role in this process [12]. FIE-HRMS cannot easily distinguish between glucose, fructose, and (for instance) galactose, so the “hexose-sugar” designation was used in this study. However, the literature suggests that the hexose sugar changing during germination is primarily glucose [35]. Correspondingly, sugar-phosphates feed into increases in pyruvate. The central metabolic pathway that is known to provide the energy for the cells and anabolic precursors for proliferation and survival is the TCA cycle [6]. However, examination of the embryo TCA cycle metabolite highlighted differences between the species. These differences were seen in elevated levels of hexose sugars and hexose sugar phosphates in Bs and BdBs compared to Bd, and this was also seen in the important TCA intermediates (fumarate, malate, and oxaloacetate). Indeed, TCA metabolites did not greatly change in Bd, and it may be that pyruvate was being diverted to lactate (which was significantly higher in Bd compared to Bs and BdBs). If the less-bioenergetic lactate pathway is more utilized during Bd germination, this could have consequences in terms of relative germinative fitness. This may also be reflected in the slightly more oxidized conditions seen in Bd, as indicated by the ratios of reduced/oxidized glutathione ratios (Figure S3). Many studies have suggested that doubling of the genome size may be associated with the increased accumulation of primary metabolites [4,5,6], especially the intermediates of TCA cycle—malate, fumarate, citrate, and succinate. This would align with our observations for BdBs, but because it was also seen in Bs (234 Mb [29], compared with that of 272 Mb for Bd [53]), it could suggest that this property was inherited from Bs in the formation of the allopolyploid. Yi et al. [50] demonstrated altered sugar metabolism alteration in *Brassicoraphanus* hybrids. There were higher levels of sugars and utilization in hybrids compared to the parental lines. These results supported similar changes observed in *A. thaliana* and rice, which shown the shift in sugar metabolism in hybrids [54,55,56] which could also be relevant to polyploidization.

In addition to bioenergetic metabolism, amino acid processing is a key aspect of germination and the expected progressive increases in their levels were seen in *Brachypodium*. The changes were similar in each species with only minor differences, e.g., glutamate levels tended to be higher in Bd. In amide processing, aminotransferases transfer the amino group from glutamate to catalyze the formation of amino acids, a notable example being asparagine synthetase which forms asparagine [57]. It is therefore of interest that asparagine is also elevated in Bd. Whatever the potential selective advantages this could represent in terms of improved amide processing, this is not a metabolomic feature of the allotetraploid embryo.

Oxalates that accumulate to high levels in wheat embryos are substrates for oxalate oxidase (a member of the germin family of proteins) to generate H_2_O_2_. Within the context of a germinating seed, the generated H_2_O_2_ could initiate programmed cell death to compromise cellular permeability barriers, and/or the peroxidative cross-linking of the cell wall. The latter would increase cell wall rigidity and counter the release of nutrients that could be important for germination in *Brachypodium* [35]. In the case of Bs and BdBs, both were shown to exhibit increased levels of oxalate and its precursor, glyoxylate, compared to Bd at 24 HAI. These increased oxalate levels were features of the embryo rather than endosperm where one might expect to detect oxalate released from the degradation of thickened cell walls (Figure 8). Thus, the increased H_2_O_2_ generation may be involved in nutrient acquisition, as seen most obviously in the increased levels of hexose sugars in Bs and BdBs.

Comparing the patterns seen with the embryo with those in endosperm, metabolites linked to sugar mobilization did not give a clear trend. Monosaccharide hexose sugars were not found to be amongst the major sources of variation between genotypes or timepoints. Furthermore, changes seen in raffinose content did not appear to have a time or genotype specific pattern (Figure S6). It may be that the limited starch in the *Brachypodium* endosperm was mobilized within 4 HAI, and this was not captured by our experimental design. Having said that, the levels of raffinose tended to be greater in Bs and BdBs. Pyruvate and the TCA metabolites—*cis*-acontiate, malate, and fumarate—were also elevated in BdBs at 4 HAI but not in Bs (Figure S6). If the cell wall is a major source of sugars, we may have seen changes in such as xylans and mannans, as well as glucose, arising from cellulose and hemicellulose polymer processing. These sugars were also not amongst the major sources of variation. However, hydroxycinnamic acids and monolignol metabolites, which are linked with cell wall modification, were prominent (Figure 9). As noted by Gullion et al. [36], the major hydroxy cinnamic acids detected were ferulic acid and para-coumarate, whilst sinapic acid was not detected. Compared to Bd, the coumarate and caffeate in Bs and BdBs were reduced whilst ferulate and 5-hydroxy/coniferyl alcohols were increased. Such alterations could be consistent with changes aiming to reinforce the cell wall after a possible rapid (i.e., <4 HAI) processing of associated sugars. Such rapid processing correlated with increased levels of, e.g., hexose sugars in the embryo (Figure 6). Therefore, this monolignol processing in Bs and BdBs may be an echo of more efficient cell wall sugar mobilization to the embryo compared to Bd. Such a feature could be an important trait of Bs that was selected for in the ancestral allotetraploidization to form BdBs. By way of comparison, whilst amino acid changes were seen within each species, which would indicate protein processing, no clear difference between the species was observed (Figure S7). Therefore, amino acid processing patterns from either Bd or Bs do not dominate that seen in BdBs.

## 5. Conclusions

The processes underlying seed germination are fundamental to plant fitness and in driving agricultural yield. These have been understandably extensively characterized in cereals but not to the same degree in other grasses. The *Brachypodium* seed is distinctive from those of domesticated temperate cereals, with thick cell walls likely to be the source of sugars as opposed to starch in the endosperm. Our metabolomic approach sought to characterize how metabolites were mobilized out of the endosperm to embryo. The results were consistent with a rapid mobilization for sugars out of the endosperm, with evidence for cell wall remodeling based on hydroxycinnamic acid and monolignol changes. More importantly, our comparative metabolomic approach allowed us to compare these changes in the seeds of Bd and Bs, the parents of the allotetraploid BdBs. In the Mediterranean basin, Bs is comparatively rare compared to Bd and BdBs, so the importance of Bs as a parent is unclear. In our metabolomic studies, the Bs metabolome was most similar to that of BdBs, suggesting that the Bs metabolome was retained during the allotetraploidization process. The conserved Bs metabolome is likely to reflect more efficient mobilization of sugars out of the endosperm than occurs in Bd, and this could have been instrumental in driving an environmentally fit BdBs species. As our observations were based on single examples of each species (albeit established community standards), they require confirmation by more expansive studies involving more genotypes including de novo hybrids. Finally, a logical extension of our approach is to consider what the metabolomic contributions of Bd are in deriving BdBs. If not in germination, it may be that Bd is important in conferring other traits, such as stress tolerance, to BdBs.

## Data Availability

The data presented in this study are available as supplementary files.

## Supplementary Materials

The following are available online at https://www.mdpi.com/article/10.3390/cells10040828/s1. Table S1: Metabolomic matrices for the *Brachypodium* species embryo and endosperm. Figure S1: Pathway enrichment metabolic pathway analysis of major sources of metabolite variation in (A) *Brachypodium distachyon* (Bd), (B) *B. stacei* (Bs), and (C) *B. hybridum* (BdBs) embryos. Figure S2: Discriminatory bioenergy metabolites detected in *Brachypodium* embryos during germination. Figure S3: Reduced (GSH) and oxidized (GSSG) glutathione levels in *Brachypodium* embryos during germination. Figure S4: Discriminatory amino acid metabolites detected in *Brachypodium* embryos during germination. Figure S5: Pathway enrichment metabolic pathway analysis of major sources of metabolite variation in (A) *Brachypodium distachyon* (Bd), (B) *B. stacei* (Bs), and (C) *B. hybridum* (BdBs) endosperms. Figure S6: Discriminatory bioenergy metabolites detected in *Brachypodium* endosperm during germination. Figure S7: Discriminatory amino acid metabolites detected in *Brachypodium* embryos during germination.
