# Supplementary material for: Allotetraploidization in Brachypodium May Have Led to the Dominance of One Parent’s Metabolome in Germinating Seeds
Source: Cells. 2021 Apr 7;10(4):828. doi: 10.3390/cells10040828 (PMC8067749; doi:10.3390/cells10040828)
Supplement: Supplementary file 1 [file cells-10-00828-s001.zip › cells-1146648 supplementary revised/R1-cells-1146648-supplementary figures S1-S7.pdf]

cells

MDPI

Supplementary Materials

Article Title: Allotetraploidisation in *Brachypodium* May Have Led to the Dominance of One Parent's Metabolome in Germinating Seeds

Authors: Aleksandra Skalska, Elzbieta Wolny, Manfred Beckman, John H. Doonan, Robert Hasterok and Luis A. J. Mur

The following Supplementary Materials are available for this article: **Supplementary Figures S1-S7**

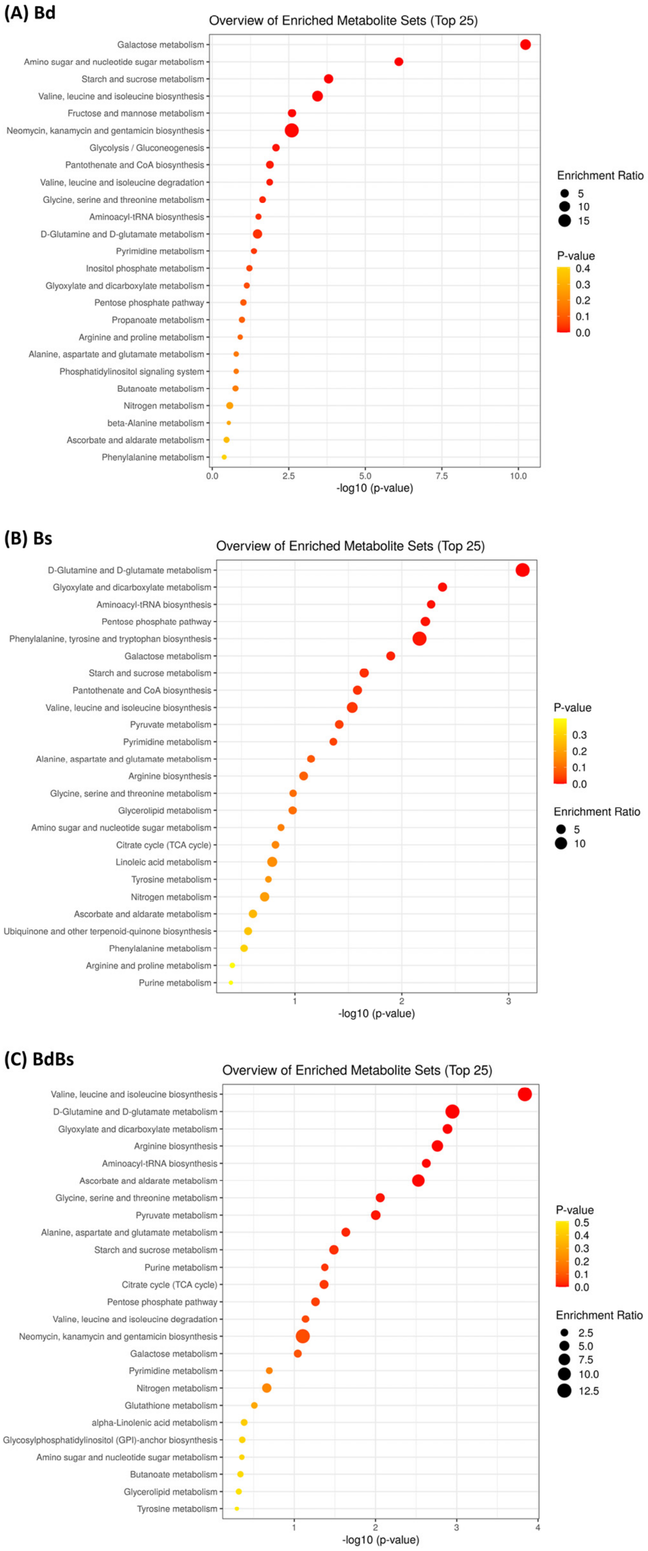

Figure S1. Pathway enrichment metabolic pathway analysis of major sources of metabolite variation in **(A)** *Brachypodium distachyon* (Bd), **(B)** *B. stacei* (Bs) and **(C)** *B. hybridum* (BdBs) embryos.

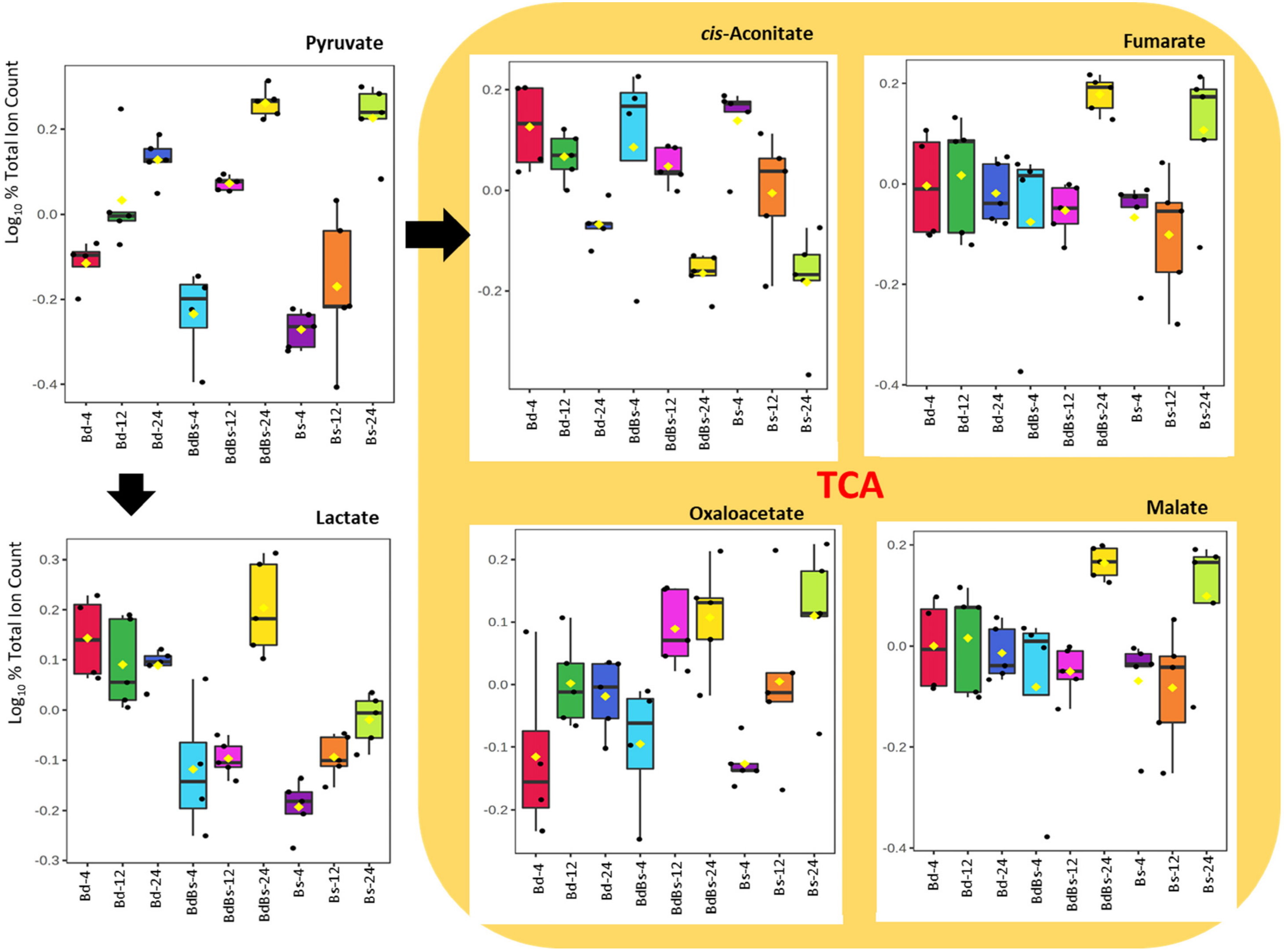

Figure S2. Discriminatory bioenergy metabolites detected in *Brachypodium* embryos during germination. Metabolites are arranged in accordance with the KEGG citrate cycle metabolism map (https://www.genome.jp/kegg-bin/show_pathway?map00020, access date, 28 February 2021). Data show box and whisker comparison of *B. distachyon* Bd21 (Bd), *B. stacei* ABR114 (Bs) and *B. hybridum* ABR113 (BdBs) at 4, 12 and 24 hours after imbibition (HAI).

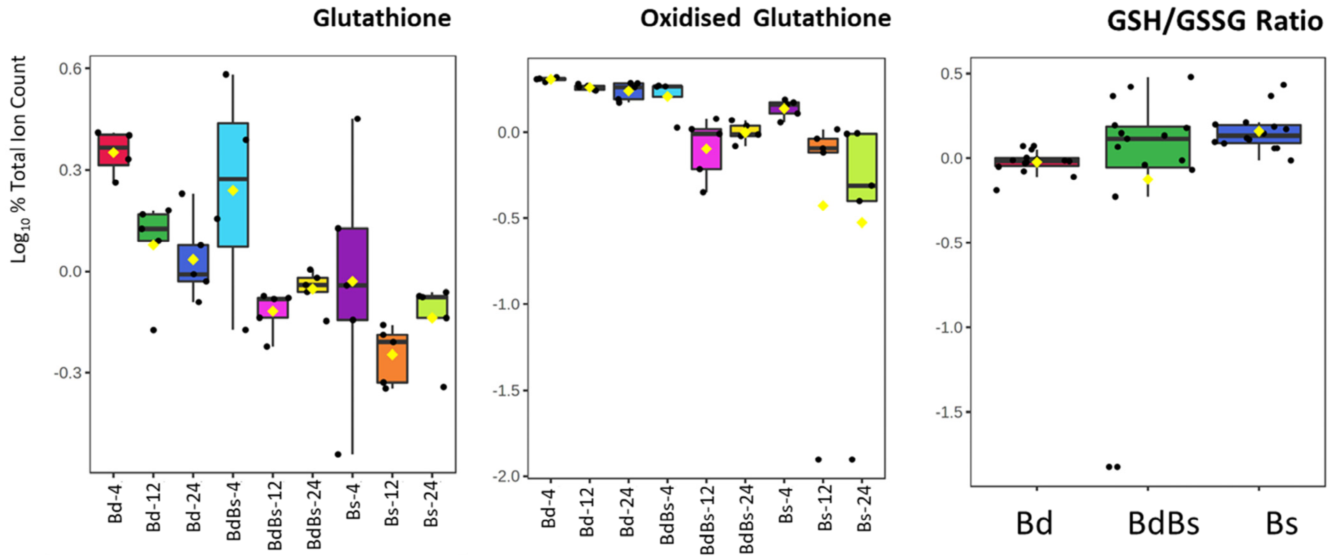

Figure S3. Reduced (GSH) and oxidised (GSSG) glutathione levels in *Brachypodium* embryos during germination. Data show box and whisker comparison of *B. distachyon* Bd21 (Bd), *B. stacei* ABR114 (Bs) and *B. hybridum* ABR113 (BdBs) at 4, 12 and 24 hours after imbibition (HAI). Overall redox status is indicated for each species based on the ratio of GSH to GSSG.

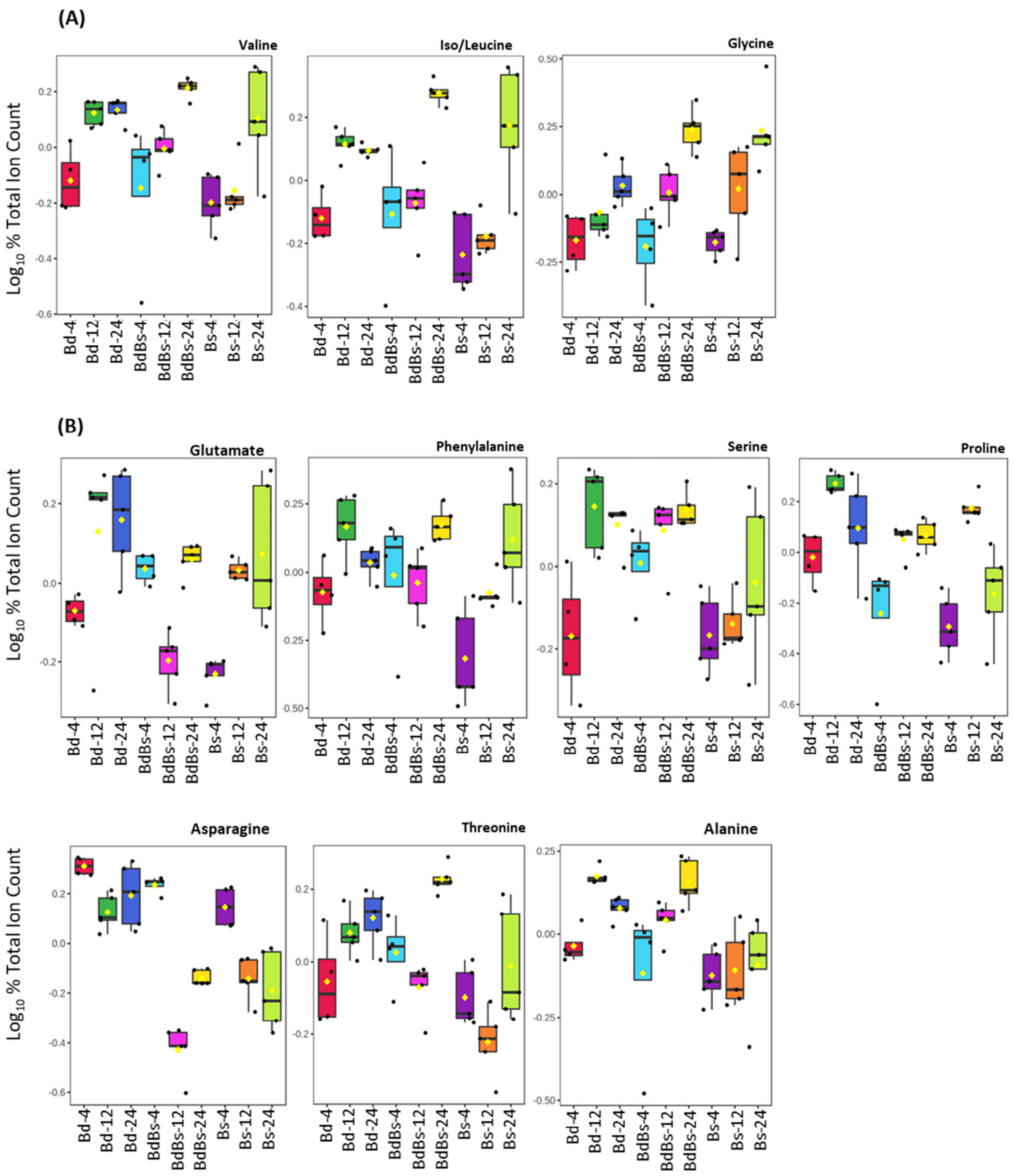

Figure S4. Discriminatory amino acid metabolites detected in *Brachypodium* embryos during germination. Data show box and whisker comparison of *B. distachyon* Bd21 (Bd), *B. stacei* ABR114 (Bs) and *B. hybridum* ABR113 (BdBs) at 4, 12 and 24 hours after imbibition (HAI).

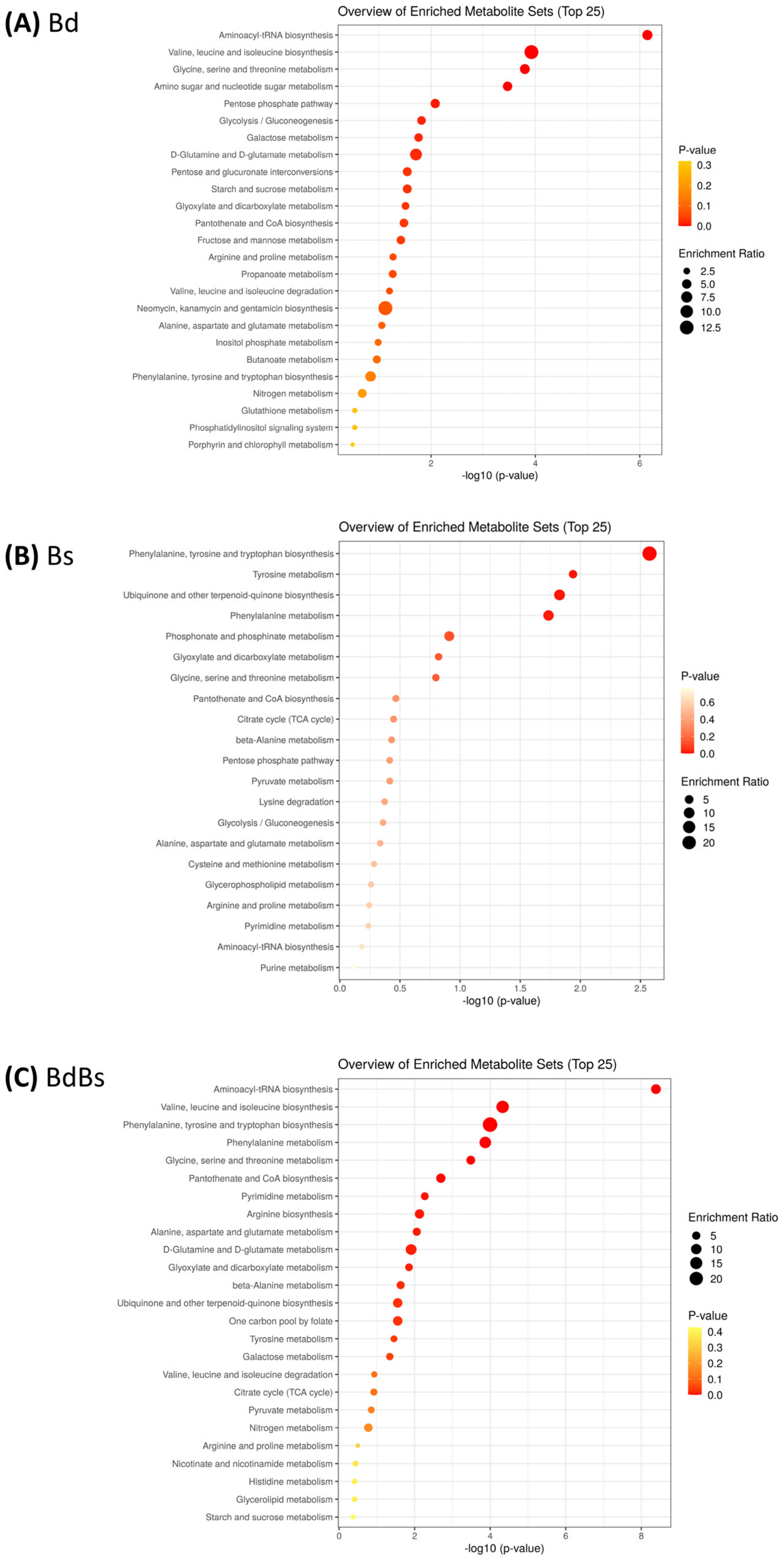

Figure S5. Pathway enrichment metabolic pathway analysis of major sources of metabolite variation in **(A)** *Brachypodium distachyon* (Bd), **(B)** *B. stacei* (Bs) and **(C)** *B. hybridum* (BdBs) endosperms.

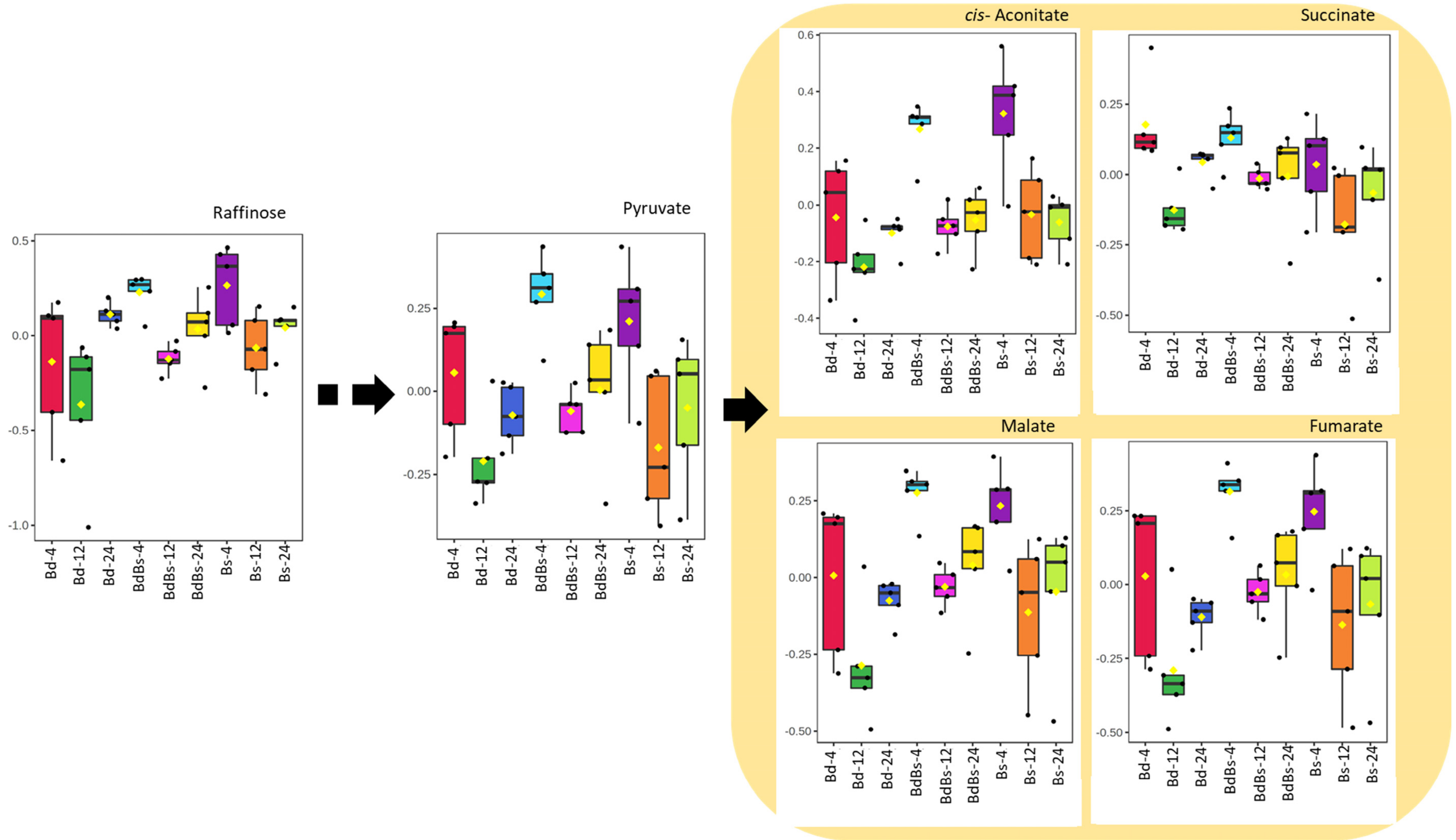

Figure S6. Discriminatory bioenergy metabolites detected in *Brachypodium* endosperm during germination. Metabolites are arranged in accordance with the KEGG citrate cycle metabolism map (https://www.genome.jp/kegg-bin/show_pathway?map00020, access date, 28 February 2021). Data show box and whisker comparison of *B. distachyon* Bd21 (Bd), *B. stacei* ABR114 (Bs) and *B. hybridum* ABR113 (BdBs) at 4, 12 and 24 hours after imbibition (HAI).

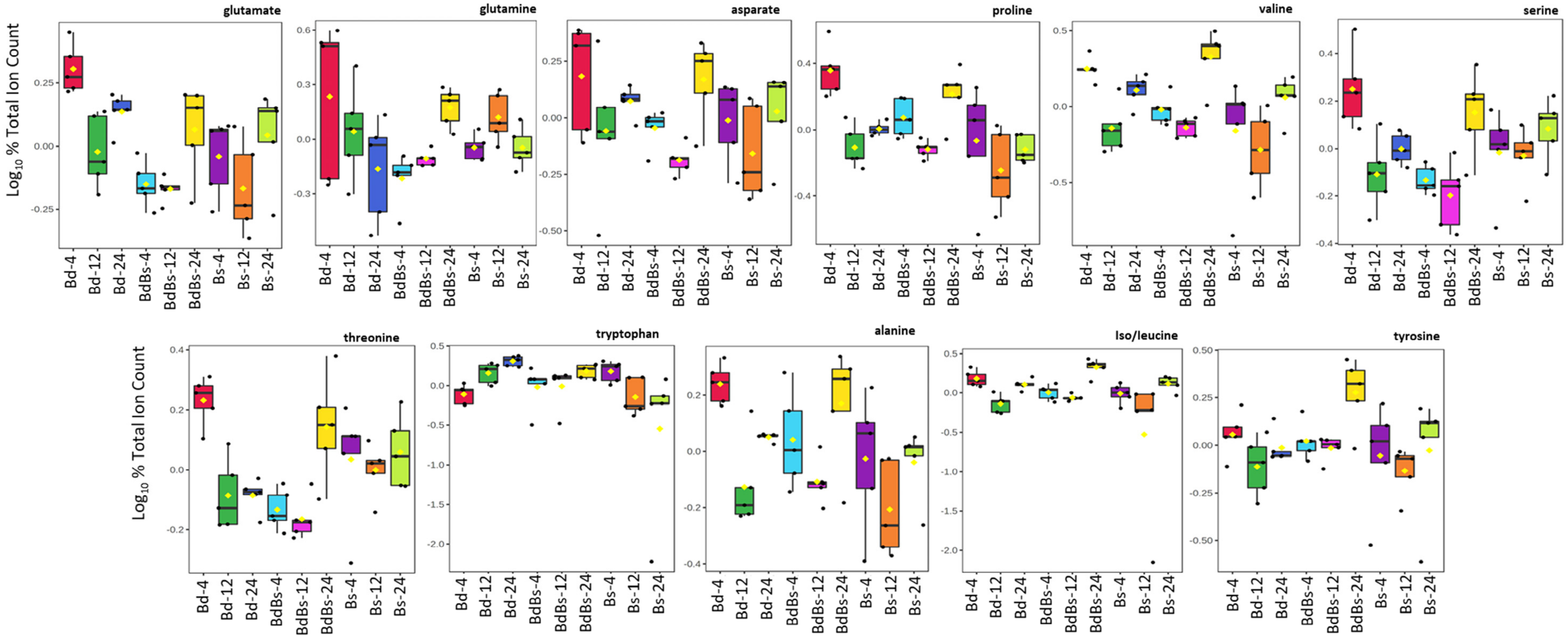

Figure S7. Discriminatory amino acid metabolites detected in *Brachypodium* embryos during germination. Data show box and whisker comparison of *B. distachyon* Bd21 (Bd), *B. stacei* ABR114 (Bs) and *B. hybridum* ABR113 (BdBs) at 4, 12 and 24 hours after imbibition (HAI).
